# Self-stigma in a Tunisian population of stabilized outpatients with schizophrenia

**DOI:** 10.1192/j.eurpsy.2024.1575

**Published:** 2024-08-27

**Authors:** S. Ajmi, M. Bouhamed, R. Ouali, R. Masmoudi, I. Feki, R. Sallemi, J. Masmoudi

**Affiliations:** Psychiatry A, Hedi Chaker University Hospital, Sfax, Tunisia

## Abstract

**Introduction:**

The internalized stigma associated with mental illness is considered as an additional burden faced by people with mental disease. Among mental illnesses, schizophrenia is considered as the most stigmatizing.

**Objectives:**

To Assess the level of stigma in a sample of people with schizophrenia

**Methods:**

This is a cross-sectional and descriptive study carried out on 72 stabilized patients followed at the post-cure psychiatry consultation ‘A’ at the CHU Hedi Chaker in Sfax diagnosed with schizophrenia according to the DSM 5 criteria. Socio-demographic and clinical data were collected using a pre-established sheet

We used The Internalized Stigma of Mental Illness (ISMI) scale to assess internalized stigma

**Results:**

The mean age of the patients in our study was 46.83 ± 11.6 years, with a sex ratio (M/F) of 2.

They were single in 48.5%, unemployed in 69.4%. Their level of education did not exceed primary school in 44.4% and their socio-economic level was low in 63.9%. 2% of the patients had no somatic history and 36.1% had a history of attempted suicide.

The median for the total ISMI score was 2.45, which corresponded to the absence of strong stigma. The median of the subscales was distributed as follows: 2 for the level of alienation, 2.28 for stereotype endorsement, 2.4 discrimination experience, 2.36 for social withdrawal and 2.60 for stigma resistance.

In our study, 45.8% of patients reported experiencing high levels of self-stigma (total score>2,5).

**Conclusions:**

Our study found levels of self-stigma in individuals with schizophrenia that align with previous research, suggesting that schizophrenia-related stigma is a global phenomenon unaffected by factors such as origin or ethnicity.

**Disclosure of Interest:**

None Declared

